# Captivity Shifts Gut Microbiota Communities in White-Lipped Deer (*Cervus albirostris*)

**DOI:** 10.3390/ani12040431

**Published:** 2022-02-11

**Authors:** Bin Li, Hongmei Gao, Pengfei Song, Chenbo Liang, Feng Jiang, Bo Xu, Daoxin Liu, Tongzuo Zhang

**Affiliations:** 1 Key Laboratory of Adaptation and Evolution of Plateau Biota, Northwest Institute of Plateau Biology, Chinese Academy of Sciences, Xining 810001, China; libin@nwipb.cas.cn (B.L.); gaohm@nwipb.cas.cn (H.G.); pfsong@nwipb.cas.cn (P.S.); jiangfeng@nwipb.cas.cn (F.J.); xubo@nwipb.cas.cn (B.X.); liudx@qhu.edu.cn (D.L.); 2 College of Life Sciences, University of Chinese Academy of Sciences, Beijing 100049, China; 3 Qinghai Provincial Key Laboratory of Animal Ecological Genomics, Xining 810001, China; 4 State Key Laboratory of Plateau Ecology and Agriculture, Qinghai University, Xining 810016, China; liangchengbo2690@outlook.com

**Keywords:** white-lipped deer, gut microbiota, captivity, assembly process

## Abstract

**Simple Summary:**

Captivity is a common conservation method for endangered animals. However, a growing number of recent studies have shown that some animals in captivity might be in sub-health condition. The gut microbiota has been described as a complex, interactive internal system that has effects on diseases of the host with many interactions, and the occurrence of certain diseases is accompanied by changes and disorder of gut microbiota. We used16S rRNA sequencing technology and a mathematical model to find differences in gut microbiota composition and assembly processes. The results show that captivity might be unfavorable for white-lipped deer by shifting the gut microbiota composition and assembly process.

**Abstract:**

White-lipped deer (*Cervus albirostris*) is a nationally protected wild animal species in China, as well as a unique and endangered species, according to the International Union for Conservation of Nature (IUCN) Red List. Captivity may alleviate the pressure from poaching and contribute to the repopulation and conservation of the population in the wild. The gut microbiota is described as a complex, interactive internal system that has effects on diseases of the host, with many interactions. However, the influence of captivity on the composition and assembly process of gut microbiota in white-lipped deer is unclear. This study applied high-throughput 16S rRNA sequencing technology to determine differences in the gut microbiota between captive (CW) and wild (WW) white-lipped deer. We used the null model, neutral community model, and niche width to identify whether captivity affects the composition and assembly process of gut microbiota. The results show that WW has a higher number of Firmicutes and a lower number of Bacteroidetes compared with CW at the phylum level, and it has more opportunistic pathogens and specific decomposition bacteria at the genus level. Principal coordinate analysis also indicated significant differences in the composition and function of gut microbiota in CW and WW. Moreover, the results reveal that captivity shifts the ecological assembly process of gut microbiota by raising the contribution of deterministic processes. In conclusion, our results demonstrate that captivity might potentially have an unfavorable effect on white-lipped deer by continually exerting selective pressure.

## 1. Introduction

White-lipped deer (*Cervus albirostris*), which belong to the Cervidae family, live above 3500 m, on average, and even on bare alpine rock (5700 m) [1,2]. It is a rare endangered species that is distinctively distributed in the Qinghai–Tibet Plateau, China [3,4]. In the last century, white-lipped deer were poached and privately caught due to the special economic value of their antlers. Meanwhile, the exponential growth of livestock competing for resource of meadow also led to a serious decrease in the of population [5,6]. Due to the rapid decrease, the species was listed as vulnerable (VU) on the International Union for Conservation of Nature (IUCN) Red List, and as endangered (EN) on the Red List of China’s Vertebrates [7,8]. To prevent poaching for antlers, reduce the demand for antlers and blood, and conserve the population, people started domesticating and breeding the deer in captivity in the 1960s to address their severely decreasing numbers [5].

Complex gut microorganic systems that inhabit mammalian intestines consist of massive amounts of microorganisms. The gut microbiota is a complex product of the long-term evolution of host and microorganisms [9]. Recent investigations have shown that the gut microbiota not only is a part of the host, but also has a significant influence on the host’s health, for example, boosting immunity, digestion, metabolism, and enteroendocrine [10,11,12,13]. The abundant food of herbivores needs a specific microbiome for disintegration and digestion [14,15]. At the same time, the complex and flexible micro-ecosystem of gut microbiota can be influenced by multiple environments and host genotypes [16]. For instance, with a change in the diet, the function, diversity, and relative abundance of certain microbes in the gut microbiota can change, and a diet-induced loss of microbial function and diversity can increase the risk of diversity loss and extinction by amplification over generations [17,18]. Captive reproduction and ex situ conservation are direct, effective strategies for conserving endangered animals. Captive individuals can also be reintroduced to the wild for the conservation and revival of the wild deer population. However, a growing number of studies have shown that some animals in captivity might be in a sub-health condition, including forest musk deer (*Moschus berezovskii*), panda (*Ailuropoda melanoleuca*), Namibian cheetah (*Acinonyx jubatus*), and Chinese pangolin (*Manis pentadactyla*) [19,20,21,22]. This state manifests as increased potential pathogens, decreased gut microbiota diversity, reduced function of certain gut microbiota, and high risk of disease. Li et al. studied the composition of gut microbiota in wild white-lipped deer, but the influence of captivity on intestinal flora has yet to be elucidated in captive (CW) and wild (WW) white-lipped deer [23].

The ecological process of community is usually a core subject in the field of community ecology and is generally used in microecosystems and environmental microbial systems. Deterministic and stochastic processes, based on niche-based and neutral theory, reveal the assembly processes of microflora. Deterministic processes, including environmental filtering and various biological interactions, such as competition, mutualism, predation, and facilitation, influence the patterns of community structure [24]. At the same time, stochastic processes, which can be defined as random changes in the community structure concerning species due to birth, death, immigration and emigration, spatiotemporal variation, and historical contingency, affect the assembly of microflora [24,25]. A growing body of work shows that deterministic and stochastic processes are not contradictory, but rather work concurrently to control the ecological assembly processes of microbiota communities [24,25,26,27,28]. However, the relative contributions of the two processes are changeable and diverse in different ecological systems. For example, Martínez et al. found a higher relative contribution of the deterministic process in Rag1-/- mice compared with wild-type counterparts in enteric microflora assembly [29]. Li et al. found that the deterministic process dominates the gut microbiota assembly process in high-altitude pikas (*Ochotona curzoniae*), while in low-altitude pikas the relative contribution of both processes is similar [30]. Communities of gut microbiota are not only influenced by host characteristics, but also by the external conditions, and assembly might be more complicated in some environmental microorganism communities [16]. Therefore, investigating changes in the gut microbiota assembly process along with changes in the environment has important t implications for shaping healthy intestinal bacterial communities by regulating diets.

In the present study, we used 16S rRNA gene amplicon technology for high-throughput sequencing on the Illumina NovaSeq sequencing platform. Then, we analyzed the composition and diversity in CW and WW. To explore the influence of captivity on the assembly of intestinal microbiota, we used the modified stochasticity ratio (MST) to quantify the deterministic and stochastic processes, and the null model, neutral community model, and niche width to calculate the relative contribution of each process. The aims of this study were as follows: (i) to explore differences in the composition and diversity of gut microbiota in different environments, (ii) to analyze the microbiota in terms of significant differences and functions, and (iii) to explore the role of captivity in the community assembly of gut microbiota.

## 2. Materials and Methods

### 2.1. Sample Collection

Fecal samples from white-lipped deer living in the wild were obtained from the region of the San Jiangyuan Nature Reserve, Zaduo County, on the Qinghai–Tibet Plateau in January 2021. We spotted and followed deer at a distance, picking up fresh and shiny droppings along the way. One dung mound was regarded as being created by one deer. A total of 17 fresh fecal samples of wild deer with natural defecation were collected. The average temperature of Zaduo County in January is −16 °C, which allowed the freshness of the wild fecal samples to be retained as much as possible. Fecal samples from captive white-lipped deer were collected at the Qinghai–Tibet Plateau Wild Animal Park in January 2021. In total, 7 fecal samples were collected, and none of the animals had received antimicrobial drugs within the last 3 months. All samples from wild and captive animals were collected using polyethylene (PE) gloves, which were then replaced to avoid cross-contamination, and samples were stored at −80 °C with liquid nitrogen. All sample collection processes were performed according to the requirements of the Ethical Committee for Experimental Animal Welfare of the Northwest Institute of Plateau Biology, Chinese Academic of Science.

### 2.2. DNA Extraction and Illumina Sequencing

The total genome DNA of samples was extracted by the cetyltrimethylammonium bromide (CTAB) method. DNA concentration and purity were monitored on 1% agarose gels, then diluted to 1ng/µL with sterile water. Subsequently, we amplified a distant region (V3-V4) of 16S rRNA from fecal microbiota by polymerase chain reaction (PCR), using specific primer 341F-806R (341F: 5′-CCTAYGGGRBGCASCAG-3′, 806R: 5′-GGACTACNNGGGTATCTAAT-3′) and barcodes. All PCR reaction mixtures contained 15µL of Phusion^®^ High-Fidelity PCR Master Mix (New England Biolabs, Ipswich, Britain), 0.2µM of each primer, and 10ng target DNA, and cycling conditions consisted of the first denaturation step at 98 °C for 1 min, followed by 30 cycles at 98 °C for 10 s, 50 °C for 30 s and 72 °C for 30 s and a final extension at 72 °C for 5 min. All PCR products were mixed with an equal volume of 1X loading buffer (containing SYB green) and electrophoresis was performed on 2% agarose gel for DNA detection. The PCR products were mixed in equal proportions, and then a Qiagen Gel Extraction Kit (Qiagen, Dusseldorf, Germany) was used to purify the mixed products. Sequencing libraries were generated with the NEBNext^®^ Ultra™ IIDNA Library Prep Kit (cat no. E7645), and their quality was evaluated with a Qubit@ 2.0 fluorometer (Thermo Scientific, Waltham, MA, USA) on the Agilent Bioanalyzer 2100 system. Finally, sequencing was performed on an Illumina NovaSeq platform and 250 bp paired-end reads were generated, and all raw data were submitted to the National Center for Biotechnology Information (NCBI) Sequence Read Archive (SRA) under accession ID: PRJNA788557.

### 2.3. Statistical Analysis

We used QIIME2 (version 2020.06) to execute the bulk bioinformatics analysis process. All raw reads were utilized to obtain reads by cutting off unique barcodes and primer sequences, then the resulting reads were merged using FLASH (version 1.2.11, http://ccb.jhu.edu/software/FLASH/, accessed on 2 October 2021), and fastp software (version 0.20.0) was used to perform quality filtering [31]. Subsequently, we used the Divisive Amplicon Denoising Algorithm 2 (DADA2) plugin of QIIME2 to denoise the sequence according to the manual, which include removing low-quality sequences, filtering out noisy, chimeric, and singleton sequences, and correcting errors in edge sequences [32,33]. Then, we obtained the output files from DADA2 including a feature table (raw ASVs table) and representative ASVs sequences. 

The Reference Sequence Annotation and Curation Pipeline (RESCRIPt) plugin in QIIME2 was used to annotate the sequence based on the compatible SILVA SSU Ref NR 99 database (version 138.1) [34]. Then, the q2-feature-classifier plugin, based on the classify-sklearn algorithm, was used for taxonomic classification. We also filtered ASVs containing mitochondria, chloroplasts, or archaea. Sequences that were unable to be allocated to the corresponding taxonomic level were recognized as a new category named “unclassified”. Then, we used the lowest sequencing depth of all samples (depth = 43,890) to normalize the raw ASVs in order to eliminate sample heterogeneity for further analysis. 

Alpha diversity indices (Chao, Shannon–Wiener, Simpson, and observed ASVs) were used to evaluate gut microbiota diversity and richness, and beta diversity distance matrices were calculated using QIIME2 software. Principal coordinate analysis (PCoA) based on Bray–Curtis, weighted and unweighted UniFrac distances was performed using the vegan package and displayed using the ggplot2 package in R software (version 4.1.1) [35,36]. Additionally, analysis of similarities (ANOSIM) based on the Bray–Curtis and weighted, and unweighted UniFrac distances was used to determine the significance of difference in two groups. Linear discriminant analysis effect size (LEfSe) with LDA score >1 and *p* < 0.05 was used to find statistically significant biomarkers in abundance between groups at different levels. STAMP was used to find significant differences between two groups of bacteria at the phylum and genus level and Wilcoxon test was used to verify significant differences [37]. All DNA extraction, Illumina NovaSeq sequencing, and QIIME2 processes were performed by Novo gene Biotech Co., Ltd. 

To study the influence of captivity on the ecological assembly process of gut microorganisms between captive and wild white-lipped deer, we used the modified stochasticity ratio (MST) to reflect the contributions of stochastic and deterministic assembly processes and quantified the stochasticity of ecological processes in gut microbiota by comparing the values between the two groups [38]. This was evaluated on the basis of 30,000 simulations using the NST (normalized stochasticity ratio) package in R version 4.1.1. Then, we used the null model to test clustering or overdispersion of gut microbiota communities by examining the deviation of each observed metric from the average of the null model (C-score). The C-score was evaluated based on 30,000 simulations using the sequential swap randomization algorithm with the EcoSimR package in R version 4.1.1 [39,40]. Further, a neutral community model was used to estimate the effects of the stochastic process on gut microbiota community assembly by non-linear least-squares generating the best fit between frequency of ASV occurrence and their relative abundance [41]. Finally, to investigate the relative effects of the deterministic and stochastic processes on gut microbiota communities, we calculated Levins’ niche breadth using the spss package in R [42]. The computation of model and niche breadth was performed with R version 4.1.1. 

## 3. Results

### 3.1. Summary of High-Throughput Sequencing 

**Figure 1:**
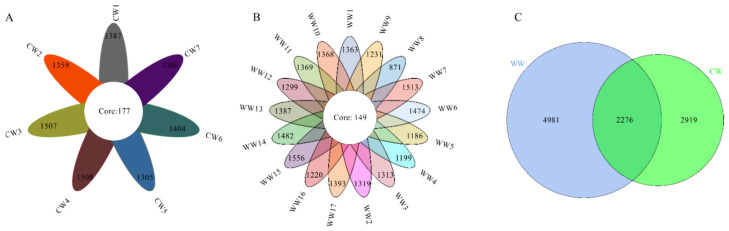
Venn and flower diagrams analysis of shared ASVs. Flower diagram shows number of ASVs that were shared (in the center) and total ASVs in each sample (in the petals) between captive (CW) and wild (WW) white-lipped deer. (**A**,**B**) Number of ASVs specific to CW and WW individuals, respectively; (**C**) number of ASVs shared by CW and WW.

The Illumina NovaSeq sequencing platform was used to amplify and detect 16S rRNA gene product sequences from fecal microbiota of 17 wild (WW) and 7 captive (CW) white-lipped deer. A total of 1,881,248 16S rRNA raw reads were obtained from 24 individuals; the V3-V4 region of the 16S rRNA gene was an average length of 410.68 bp. The raw reads were filtered by QIIME2, and a total of 1,282,048 clean reads were obtained in the present study. For each sample, 43,890–63,955 clean reads were obtained, with a median of 53,760. A Venn diagram was used to confirm the common gut microbiota in captive and wild white-lipped deer. Our result shows that 177 core ASVs were shared in the captive group and 149 ASVs were shared in the wild group (Figure 1A,B). The average number of ASVs per group was inconsistent with 7257 in the wild group, 5195 in the captive group, and 2276 ASVs shared between these two (Figure 1C).

### 3.2. Composition of the Gut Microbiota between Captive and Wild White-Lipped Deer

The rarefaction curves of wild and captive white-lipped deer fecal samples (Figure S1) show the adequacy and species richness of samples. As the sequencing depth increased, the number of observed species gradually stabilized, and there was no further significant growth or fluctuation in the two groups. Consequently, the number of samples in this study was sufficient to study the gut microbiota of wild and captive white-lipped deer.

**Figure 2:**
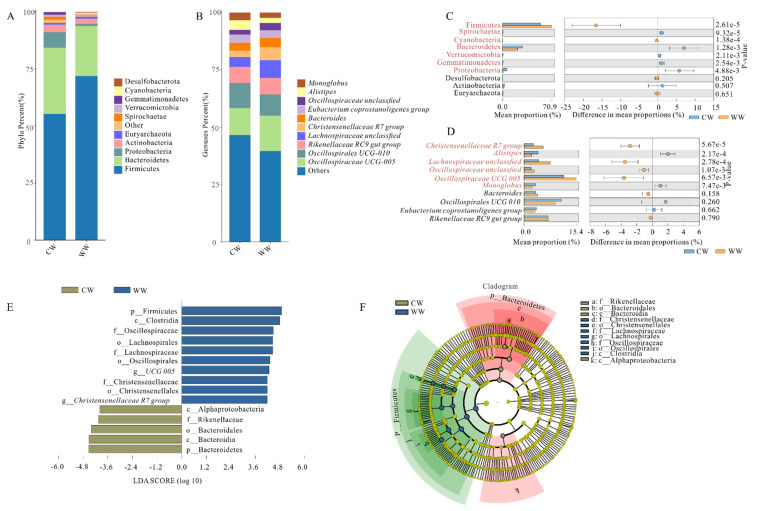
Microbiota composition of fecal samples and component difference analysis. (**A**,**B**) Bar charts show abundance of top 10 phyla and genera, respectively, between CW and WW. (**C**,**D**) Significant differences between the two groups at phylum and genus level, respectively, indicted in red (*p* < 0.05). (**E**) Result of LDA effect size determining biomarkers with statistically significant differences between groups. LDA value distribution histogram shows biomarker with statistical differences, and extent of histogram reflects degree of effect (LDA score). (**F**) Each small circle of cladogram at different levels represents a different classification; diameter is in direct proportion to relative abundance.

We detected 33 phyla, 67 classes, 172 orders, 305 families, and 637 genera in the gut microbiota communities from 24 fecal samples of white-lipped deer. Among them, 17 phyla, 27 classes, 77 orders, 137 families, 261 genera, and 309 species were shared between the groups (Figure S2). To show the relative abundance of bacterial communities more intuitively, we generated a stacked histogram of relative abundance at the top 10 phylum and genus levels between different groups (Figure 2A,B). Firmicutes comprised the most numerous microbial communities in the gut, followed by Bacteroidetes. 

We used STAMP to verify the significant difference in the top 10 phyla and genera (Figure 2C,D). In WW, the relative abundance of Firmicutes and Cyanobacteria phyla were significantly higher than in CW (*p* = 2.61e-5, 1.38e-4), in contrast, the relative abundance of Spirochaetae, Bacteroidetes, Verrucomicrobia, Gemmatimonadetes, and Proteobacteria phyla in WW was significantly lower than in CW (*p* = 9.32e-5, 1.28e-3, 2.11e-3, 2.54e-3, 4.88e-3). Meanwhile, no significant differences in Desulfobacterota, Actinobacteria, and Euryarchaeota were shown in both groups (*p* = 0.205, 0.507, 0.651). At the genus level, the relative abundance of *Christensenellaceae R7 group*, *Lachnospiraceae unclassified*, *Oscillospiraceae unclassified*, and *Oscillospiraceae UCG-005* was significantly higher compared to CW (*p* = 5.67e-5, 2.78e-4, 1.07e-3, 6.57e-3). On the contrary, CW had a significantly higher relative abundance of *Alistipes* and *Monoglobus* genera than WW (*p* = 2.17e-4, 7.47e-3). No significant differences were found in *Bacteroides*, *Oscillatoriales UCG-010*, *Eubacterium coprostanoligenes group*, and *Rikenellaceae RC9 gut group* between CW and WW (*p* = 0.158, 0.260, 0.662, 0.790). Species showing significant differences between captive and wild groups at each level were calculated by linear discriminant analysis (LDA) effect size (LEfSe) analyses. According to the results (Figure 2E,F), phylum Firmicutes, class Clostridia, family Oscillospiraceae, order Lachnospira, family Lachnospiraceae, order Oscillatoriales, genus *UCG 005*, family Christensenellaceae, order Christensenellales, and genus *Christensenellaceae R 7 group* were significantly enriched in WW. However, in CW, only phylum Bacteroidetes, class Bacteroidia, order Bacteroidales, family Rikenellaceae and class Alphaproteobacteria were significantly enriched. 

### 3.3. Variation of Gut Microbiota Diversity across Different Living Environments

**Figure 3:**
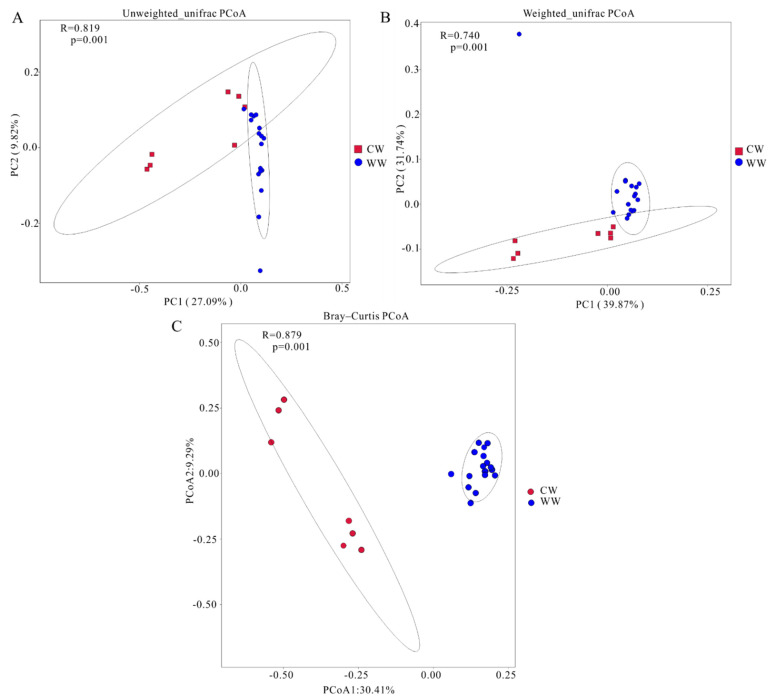
Principal coordinate analysis (PCoA) plots of gut microbiota analysis. PCoA was used to extract main elements by sorting eigenvalues and eigenvectors from multidimensional data. ANOSIM calculated by different arithmetics (R > 0 indicates that the grouping is effective). (**A**) Unweighted UniFrac; (**B**) weighted Unifrac; (**C**) Bray–Curtis. Distance of samples reflects similarity of gut microbiota community composition.

Chao, Shannon–Wiener, Simpson, and observed ASVs indices were used to evaluate gut microbiota diversity in the white-lipped deer, and the Wilcoxon test was used to analyze the significance of alpha diversity in different groups. The Shannon–Wiener index was higher in the CW group than the wild group (*p* < 0.05), while no significant differences between the groups were found by the Chao, Simpson, and observed ASVs indices (*p* > 0.05) (Figure S3). To further analyze the discrepancy between groups, we used principal coordinate analysis (PCoA) plots based on Bray–Curtis and UniFrac distance (Figure 3). Analysis of similarities (ANOSIM) based on different arithmetics reflected the significance between CW and WW (R > 0). These plots demonstrate the differences in microbiota in samples from captive and wild deer, and the distance between spots of different colors shows the similarity of the gut microbial community structure of the samples. Wild and captive white-lipped deer tended to cluster, but what is notable is the large distance between captive group samples was distant. Meanwhile, these plots have some overlaps with the UniFrac plot, but not in the Bray–Curtis plot (Figure 3).

### 3.4. Relative Importance of Deterministic and Stochastic Processesin CW and WW

**Figure 4:**
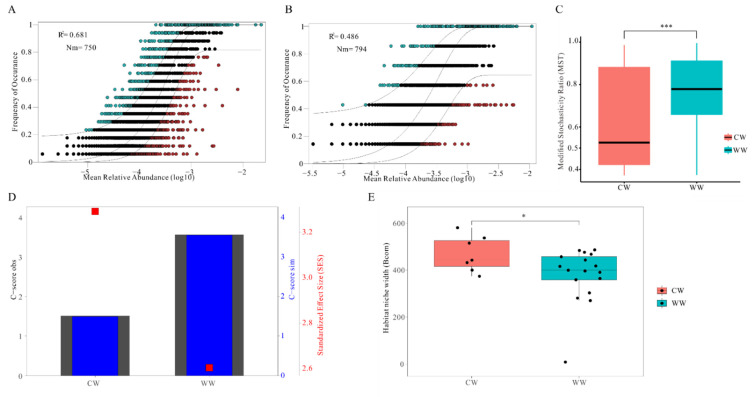
Ecological processes shaping gut microbiota communities in CW and WW white-lipped deer. (**A**,**B**) Predicted occurrence frequencies for WW and CW represent gut microbiota communities from WW groups. Solid blue line indicates best fit to neutral community model (NCM), dashed blue line indicates 95% confidence intervals around NCM prediction. OTUs that occur more or less frequently than predicted by NCM are shown in green and red, respectively. R^2^ represents fit to this model. (**C**) Evaluation of the relative significance of determinate and stochastic process between CW and WW. If modified stochasticity ratio (MST) >0.5, deterministic processes dominate; if MST < 0.05 stochastic process dominate (Not significant, *p* > 0.05; * 0.01 < *p* < 0.05; *** *p* < 0.01). (**D**) C-score index using null models. Values of observed C-score ((C-score obs) = simulated C-score (C-score sim)) indicate random co-occurrence patterns. Standardized effect sizes -2 and > 2 represent aggregation and segregation, respectively. (**E**) Comparison of mean habitat niche breadth for CW and WW groups (Not significant, *p* > 0.05; * 0.01 < *p* < 0.05; *** *p* < 0.01).

The ecological process of the deterministic and stochastic processes in CW and WW groups was evaluated by the modified stochasticity ratio (MST) (Figure 4C). The distribution of MST in both groups exceeded the threshold value (0.5) and was significantly higher in the WW group than the CW group. The neutral community model showed the relationship between the occurrence frequency and relative abundance of ASVs (Figure 4A,B). The CW group (48.6%) had a lower relative share of the stochastic process than the WW group (68.1%). All gut microbiota communities showed significantly wider niche breadth in the CW group than in the WW group (Figure 4E). Notably, the C-score reflected that the CW group had a higher standardized effect size (SES), which indicates the raised importance of deterministic processes in the ecological process of gut microbiota (Figure 4D). Meanwhile, C-score obs (observations) and C-score sim (simulation) values were similar in both groups. 

## 4. Discussion

### 4.1. Captivity Changes the Composition of Gut Microbiota 

Composition results show that Firmicutes and Bacteroidetes were the most dominant (>84%) phyla in all samples, in agreement with the results of research in herbivores, including, sika deer (*Cervus nippon*), bharal (*Pseudois nayaur*), Tibetan wild ass (*Equus kiang*) and forest musk deer [19,43,44,45]. In contrast, the relative abundance of Firmicutes was prominently higher in the WW group than in the CW group (*p* < 0.01), and the abundance of Bacteroidetes was higher in the CW group than the WW group (*p* < 0.01) (Figure 2A,B). Previous research indicated that Firmicutes play an important role in digestion absorption and the metabolism of protein and other nutrients and yields some beneficial metabolites [46,47,48]. For example, Firmicutes are the primary cellulose decomposition bacteria in herbivores and can yield volatile fatty acids by the pathway of cellulose degradation to be utilized in the host gut [49,50]. Bacteroidetes is an opportunistic pathogen that specializes in the degradation of protein and carbohydrates in intestinal digestion and absorption processes [51]. However, it is also connected to the release of the polymer-degrading enzymes targeting host cellular components in the proteolysis process [51]. An increase in Proteobacteria phylum in captive white-lipped deer might potentially be a mark of dysbiosis in the gut microbiota [52]. Notably, the phylum Gemmatimonadetes was solely discovered only in the captive group and not the wild group (Figure 2A). Bay et al. found that Gemmatimonadetes are capable of aerobic methanotrophy and can use methane hydrogen and acetate as energy sources [53]. Increased aerobic methanogens in the captive group shows that captive individuals are more dependent on methane metabolic pathways, while the wild group is deficient in aerobic methanogens. It might be attributed to a decrease in digestion and excessive food in the rumen, abomasum and gut. Furthermore, the results indicate that Firmicutes/Bacteroidetes were significantly different in the two groups, with a higher ratio in the wild group. We consider the positive role of the high proportion in the accumulation of fat, intestinal energy digestion, and absorption, consequently we speculated that the captive group had weaker ability to absorb and store energy from the diet in the gut [54]. 

In addition, similar core bacterial species between CW and WW were found at the genus level. Among these species, CW had significantly higher relative abundance of *Monoglobus* and *Alistipes*. *Monoglobus* is a highly specialized bacteria for pectin degradation that might be caused by excess exposure to human environments [55]. Opportunistic pathogens in the host gut microbiota, such as *Alistipes* and *Lachnospiraceae unclassified* were increased in both groups [56,57]. This result shows that both groups have a risk of disease in different environments. Moreover, WW had higher relative abundance of *Christensenellaceae R7 group*, which plays a crucial role in increasing blood sugar and promoting obesity in the host [58].To determine the difference in diet between CW and WW groups, we tested the composition of nutritive substances of industrial food at the Qinghai–Tibet Plateau Wild Animal Park and sere grass, collected in January 2020 (Table S1). The results show that industrial diet food has more protein, fat, total sugar, energy, and water, and less crude fiber compared with sere grass. Long-term captivity changes the composition of gut microbiota in white-lipped deer, which manifests in increase and decrease in certain bacteria and reduced function of digestion and absorption. Therefore, we infer that the increase in bacteria by digesting carbohydrate, protein, and pectin might be caused by excessive long-term intake of industrial food with high protein, fat, total sugar, and energy, which could also lead to a decline in certain gut microbiota functions. 

### 4.2. Alpha and Beta Diversity in Gut Microbiota

The Shannon and Simpson indices show species richness and evenness, the Chao index is used to estimate species richness, and observed ASVs represents the number of ASVs. Alpha diversity results show that the Shannon index was higher in captive than wild deer (0.01 < *p* < 0.05), but there was no significant variation in other alpha diversity indices of gut microbiota between CW and WW (*p* > 0.05). Therefore, the alpha diversity indices of the two groups were considered to be non-significant (*p* < 0.05). This result surprised us since it was beyond our prediction, because many previous studies had shown that the alpha diversity of gut microbiota in wild populations is higher than that in captive animals, for example, Tibetan wild ass, bharal, Tibetan sheep, and yak (*Bos mutus*) [44,45,59,60,61]. Nevertheless, it is in accord with an investigation of gut microbiota in forest musk deer [19]. Then, we found that captivity might increase the alpha diversity of gut microbiota in most Cervidae, for example, sika deer, Père David’s (*Elaphurus davidianus*), white-tailed deer (*Odocoileus virginianus*), and red deer (*Cervus elaphus*). The opposite phenomenon occurs in Cervidae compared with other animals [43,62,63,64]. It may be that some environmental stresses in the wild or the special structure of stomach and intestines in these deer lead to decreased alpha diversity of gut microbes in wild deer [65]. This phenomenon needs further research to determine and verify the reasons. Meanwhile the beta diversity result revealed significant differences in the composition and structure of microbial communities between the two groups. Considering the differences in the algorithms for Bray–Curtis and UniFrac distances, the overlaps in UniFrac PCoA plots showed certain similarities in the evolutionary classification of gut microbiota in the two groups.

### 4.3. Captivity Mediates the Ecological Assembly Process of Gut Microbiota Communities

Our results revealed that captivity has a great influence on the assembly of gut microbiota in white-lipped deer. The MST result showed a significant difference in the assembly process between wild and captive deer due to changes in the relative contribution balance between deterministic and stochastic processes (Figure 4C). Although stochastic processes were shown dominate the gut microbiota community assembly in both groups, deterministic processes were found to play a more crucial role in the captive group than in the wild group. The community variation, explained using stochastic processes, declined from 68.1% in the WW group to 48.6% in the CW group (Figure 4A,B). Furthermore, the gut microbiota had a wider niche breadth in captive than wild (Figure 4E). Additionally, the C-score result revealed that the SES increased with changing diet in the CW group, which implies that the community assembly of gut microbiota was also strongly affected by deterministic processes (Figure 4D) [40]. This result was identified by an extended and experimentally testable conceptual model [66]. It occurs due to changes in selective factors of gut microbiota as succession proceeds, which results in specific physiological changes. For example, salinity can strongly affect the bacterial community composition of the soil in a desert ecosystem, imposing a stringent environmental filter as succession proceeds [67]. We considered that captivity changed the balance of deterministic and stochastic processes during the assembly of gut microbiota by imposing a strong effect of selection and filtering on the assembly process and increasing the relative contribution of deterministic processes [68,69]. 

We found that industrial food is significantly different from food in the wild, and diet can directly affect and shape gut microbes [70,71,72], whereas the wild white-lipped deer feed on sere grasses, roots, and leaves and the branches of sere trees, which are low in fat and protein and high in fiber, natural diets promote the retention of native gut microbiota in captive animals [73]. Meanwhile, while we are aware that recreating the entire wild diet for white-lipped deer in captivity may be extremely difficult, supplementing their diet with more high-fiber food may be an alternative to support intestinal microbial communities, as observed in research on white-throated woodrat (*Neotoma albigula*), sifakas (*Propithecus coquereli*) and wild-caught great tits (*Parus major*) [74,75,76]. Alternatively, captive programs should carefully adjust artificial diets to increase the amount of probiotics. The loss of specific enteral microbial functions and some bacteria in captive white-lipped deer might be detrimental for programs focused on the release animals to the wild and the salvation and management of wild animals, and the prevention of certain disease. These results not only highlight the relationship between the gut microbiota and the health of the host in white-lipped deer, but also provide a basis for protection programs for conservation of the deer in both environments.

## 5. Conclusions

The composition and abundance of intestinal bacteria in CW and WW were found to differ significantly. Long-term industrial food was supposedly the main reason, which causes obvious difference in gut microbiota between the two groups. Perennial ingestion of industrial food led to increases and decreases in certain bacteria and altered functions of gut microbiota, and might generate sub-health conditions for captive white-lipped deer. Furthermore, it may be detrimental to the salvation and conservation of populations in the wild. These findings not only contribute to the formulation of appropriate diet management and provide valid assistance for disease diagnosis and release of white-lipped deer, but are also expected to provide a reference for the ecological process of intestinal flora in a graminivorous ungulates.

## Data Availability

Sequence data are available from the Sequence Read Archive (SRA) BioProject PRJNA788557.

## Supplementary Materials

The following supporting information can be downloaded at: https://www.mdpi.com/article/10.3390/ani12040431/s1, Figure S1: Rarefaction curves of samples; Figure S2: Venn diagram of different taxa between CW and WW: Figure S3: alpha diversity index box plots; Table S1: Composition of sere grass and industrial fodder.
