# Supplementary material for: Captivity Shifts Gut Microbiota Communities in White-Lipped Deer (Cervus albirostris)
Source: Animals (Basel). 2022 Feb 11;12(4):431. doi: 10.3390/ani12040431 (PMC8868073; doi:10.3390/ani12040431)
Supplement: Supplementary file 1 [file animals-12-00431-s001.zip › animals-1587391-supplementary.pdf]

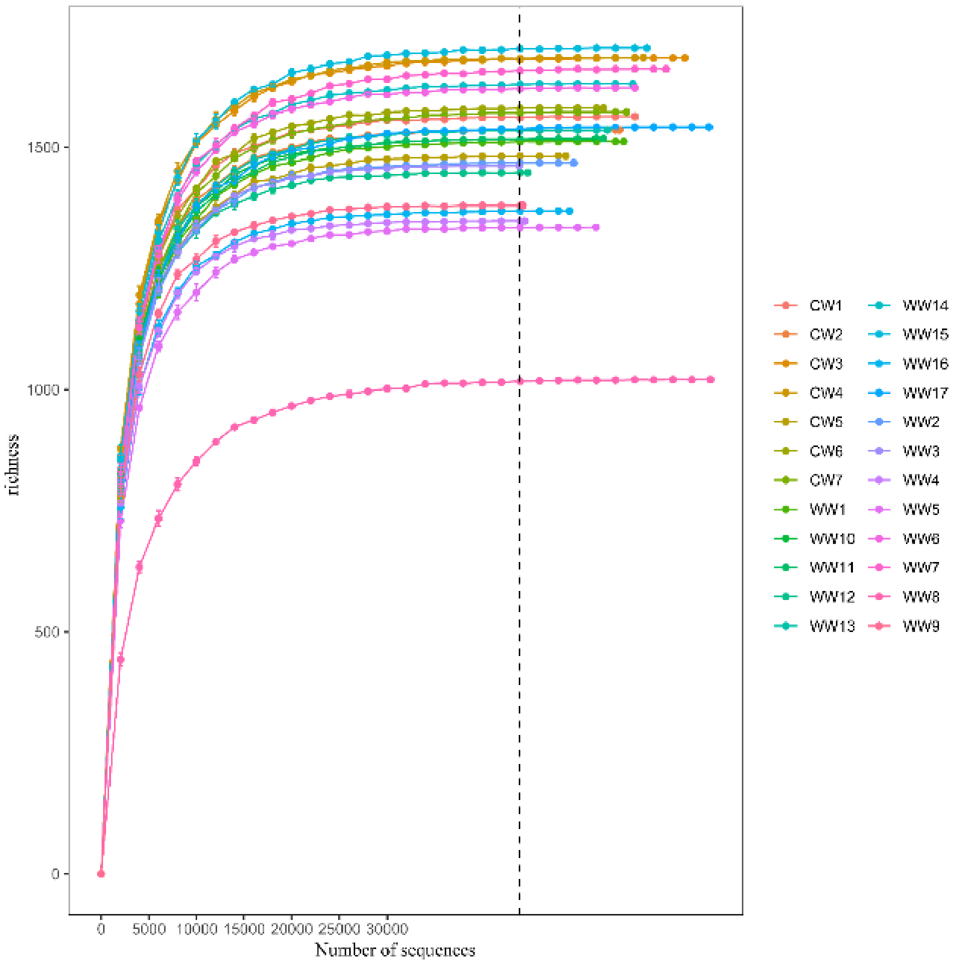

Figure S1. Rarefaction curves of samples. Richness of new ASVs arrive at peak with increasing sequencing depth, then change little.

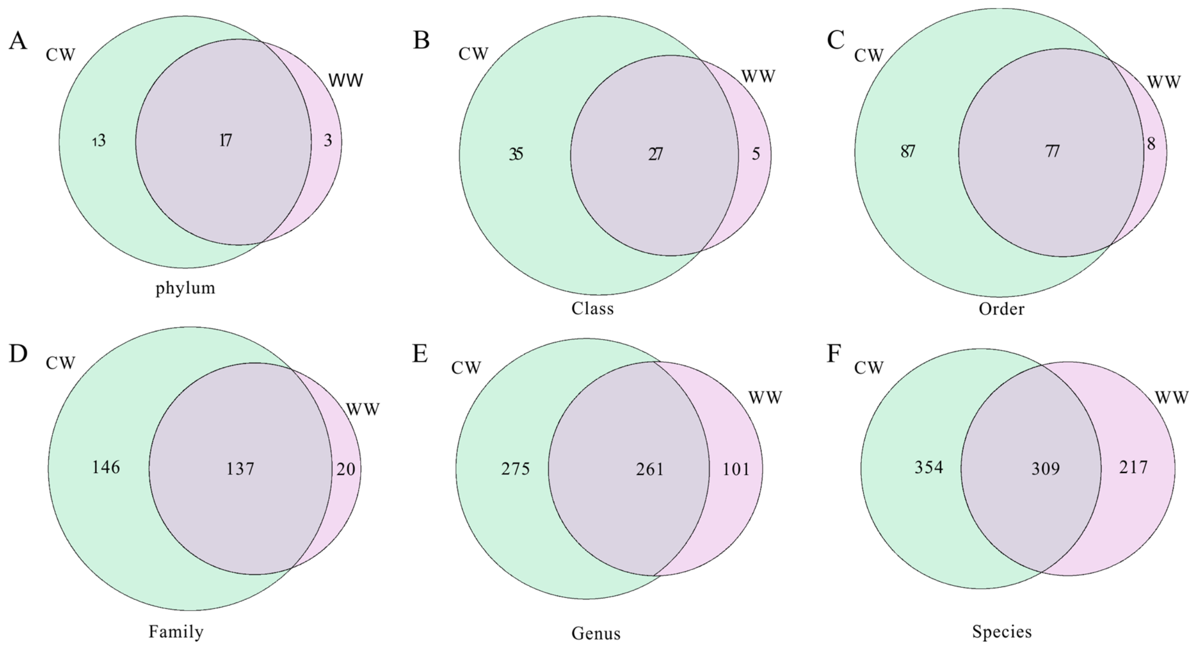

Figure S2. Venn diagrams of different taxa between Captive (CW) white-lipped deer and Wild (WW) white-lipped deer: (A) phylum level, (B) class level, (C) order level, (D) family level, (E) genus level, (F) species level.

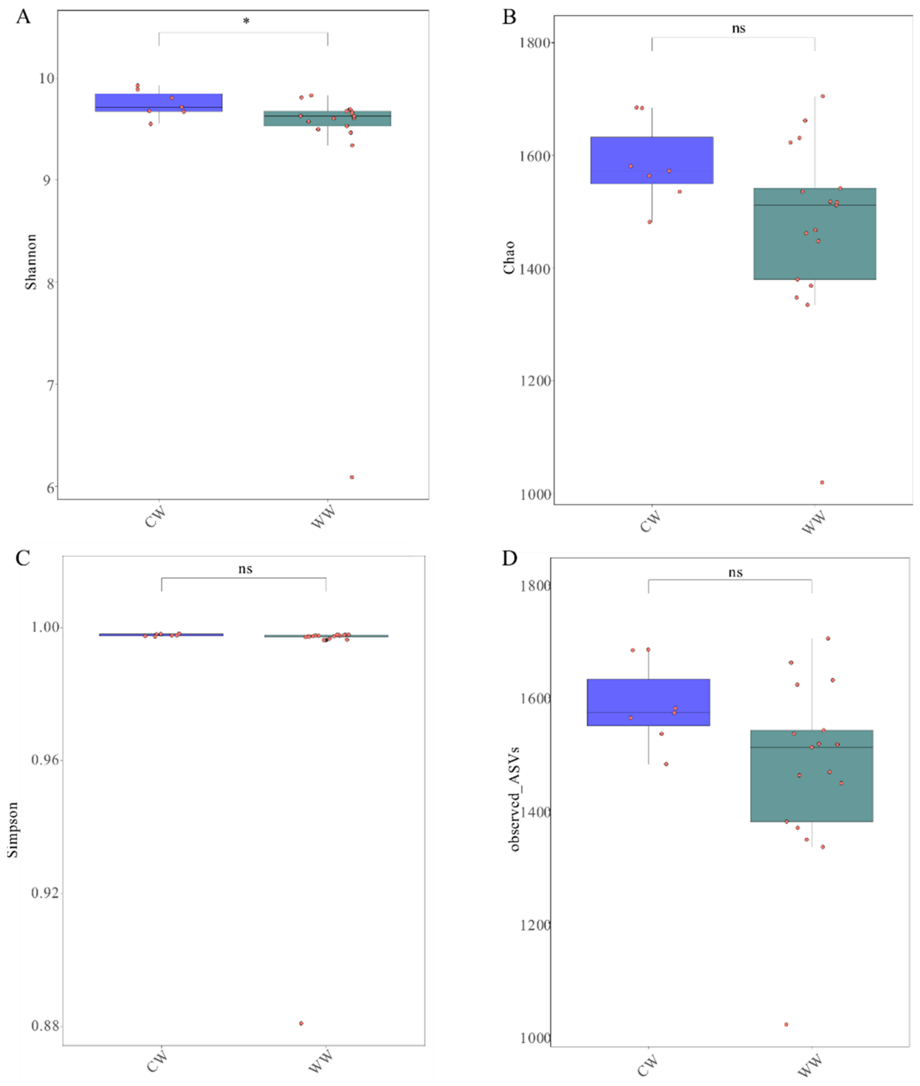

Figure S3. Alpha diversity index box plots: (A)Shannon index, (B)Chao index, (C)Simpson index, (D)observed ASVs. Wilcoxon test was used to analyze significance of alpha diversity in different groups (ns, $p > 0.05$; * $0.01 < p < 0.05$).

Table S1. Composition of sere grass and industrial fodder.

Compositions	Sere grass (oat)	Industrial fodder
Water（%）	6.9	13.0
Ash（%）	3.5	6.9
Protein（%）	3.99	19.6
Crude fat（g/kg）	20.2	34.7
Crude fiber（g/kg）	392.6	88.3
Carbohydrate（%）	44.3	48.2
Energy（KJ/100g）	1569	2437
Total sugar（%）	4.4	19.3
Na（g/kg）	1.12	3.43
P（g/kg）	0.929	4.28
Ca（g/kg）	1.99	11.0
